# Measuring hsCRP—An Important Part of a Comprehensive Risk Profile or a Clinically Redundant Practice?

**DOI:** 10.1371/journal.pmed.1000196

**Published:** 2010-02-02

**Authors:** James P. McCormack, G. Michael Allan

**Affiliations:** 1 Faculty of Pharmaceutical Sciences, University of British Columbia, Vancouver, British Columbia, Canada; 2 Faculty of Medicine, University of Alberta, Edmonton, Alberta, Canada

## Abstract

James McCormack and Michael Allan discuss issues and questions surrounding hsCRP measurements in patients.

With the publication of the JUPITER trial [1] there is now considerable interest in measuring high-sensitivity C-reactive protein (hsCRP). While treatment with rosuvastatin decreased the chance of clinically important events in the JUPITER trial, does this necessarily justify treatment based on hsCRP measurements? Here, we discuss issues surrounding hsCRP measurements in patients.

## What Is the Suggested Role for hsCRP Measurements?

The median values for hsCRP are 2.5 mg/l (American women) and 1.5 mg/l (American men) [2]. Typical recommendations are to measure hsCRP in “intermediate risk” patients to help classify them into either a higher or lower risk category [3],[4]. Intermediate risk is usually, and arbitrarily, described as either a 10%–20% [3],[4] or 5%–20% [5],[6] 10-year risk of developing coronary heart disease (CHD). It has been suggested that hsCRP levels<1, 1–3, or >3 mg/l represent lower, moderate, and higher relative risk of future heart disease, respectively.

## How Accurate Is hsCRP Measurement?

Between-subject standard deviation for hsCRP measurement is 1.7 mg/l. Within-subject standard deviation is 1.2 mg/l [7]. Clearly, within-subject standard deviation means that a patient with a reported hsCRP of 2 mg/l (moderate) when re-measured could readily be placed in a low (<1 mg/l) or high (>3 mg/l) range [7]. Some authors have therefore suggested hsCRP needs to increase or decrease by 120% to 175% before a “real” change can be considered to have occurred [8]. It has been estimated that “in order to reduce the intra-individual variation sufficiently, each subject is likely to require blood samples collected on at least 10 occasions” [9].

## Does Measuring hsCRP Add Value to Already Established Risk Factors When It Comes to Assessing CHD or Cardiovascular Disease Risk?

Several studies have confirmed that using hsCRP in addition to established risk factors (age, gender, blood pressure, cholesterol, smoking, and diabetes) does not improve the estimation of risk of cardiovascular disease (CVD) to a clinically important degree. Folsom et al. found that elevated hsCRP was associated with an increased risk of CHD (hazard rate ratio 1.19, *p*<0.001) but that it was similar to, or less important than, many other markers including D-dimer (1.36), interleukin-6 (1.28), and lipoprotein-associated phospholipase A2 (1.17) [10]. Wang et al. used C statistics to assess CVD predictive models and found age, sex, and traditional risk factors provided 0.76 value compared to the 0.77 value when ten different biomarkers (including hsCRP) were added [11]. Shah et al. also assessed the additive value of hsCRP and found that “hsCRP does not perform better than the Framingham risk equation for discrimination. The improvement in risk stratification or reclassification …is small and inconsistent” [12].

## How Does Measuring hsCRP Affect Absolute Risk Estimates of CVD in Individuals?

The Reynolds Risk Score [13] is an online calculator that incorporates hsCRP measurement with other risk factor information (age, sex, systolic blood pressure [SBP], smoking history, total cholesterol, high-density lipoprotein [HDL] cholesterol, and family history) to compute the risk (%) of heart attack, stroke, or other major heart disease in the next 10 years. We used two patients, one male and one female with a “moderate” hsCRP level (2 mg/l), and adjusted other factors so that they would have an overall absolute risk estimate of 15%—in the middle of the “intermediate” risk category. We then changed hsCRP to 0.5, 5, or 10 mg/l to see what would happen to their calculated risk (Table 1).

**Table 1:** Estimated 10 year risk of a heart attack, stroke, or other major heart disease based on the risk calculator at reynoldsriskscore.com.

Patient	hsCRP mg/l			
	0.5	2	5	10
60 y/o male^a^	13%	**15%**	16%	17%
70 y/o female^b^	12%	**15%**	17%	19%

a Nonsmoker, no family history, SBP 160 mmHg, total cholesterol of 5 mmol/l and an HDL of 1 mmol/l.

b Nonsmoker, no family history, SBP of 155 mmHg, a total cholesterol of 6 mmol/l and an HDL of 1 mmol/l.

Absolute risk estimates changed by just ±2% (male) and ±3%–4% (female). It is important to appreciate that the magnitude of these risk estimate changes are equal to or less than the confidence interval (CI) on the original risk estimate. Anderson et al. noted for the Framingham dataset that the 95% CIs for 10-year predictions of CHD of less than 10%, 10%–20%, and 30%–40% were ±1.5%–2%, ±3%, and ±15%, respectively [14]. Reynolds et al. recently calculated the CI of the estimates at 10%, 15%, 20%, and 30% thresholds to be ±4%, 5%, 6%, and 7%, respectively [15]. It seems obvious that refining such predictions by ±2%–4% by incorporating hsCRP measurements is of little clinical relevance.

Nonetheless, if we assume the changes in risk estimate using hsCRP are exact and the risk estimates are actually changed by the percentage suggested; are these changes in risk estimate clinically relevant? First, in these scenarios, one can see that none of the revised risk estimates actually end up recategorizing our particular patients into a different risk category. Second, would knowing one's risk was 17% or 13%, instead of 15% lead to a difference in the decision to consider taking a drug?

Let's assume statins produce a 25% relative risk reduction in CVD. If a patient has an absolute baseline risk of 17%, their risk, if they took a statin, would decline to 12.75%, an absolute difference of 4.25%. If their baseline risk was 15%, a statin would lower their risk to 11.25%, an absolute difference of 3.75%. In other words, in this patient, if the absolute risks were indeed different the difference in the estimate of absolute benefit would be 0.5% (4.25% minus 3.75%); a difference unlikely to change the decision to use or not use a statin.

## Don't Studies Show hsCRP Measurements Reclassify Patients into Different Risk Categories?

Ridker et al. have shown that incorporating hsCRP “reclassifies” people into different risk categories [6],[16]. For instance, 14% of women and 12% of men were recategorized from intermediate to low risk. However, others have disputed these findings and found that only 5.6% of patients would end up being reclassified [17]. What is likely happening in these “reclassification” papers is a number of patients with risks just above or below these arbitrary thresholds (say an estimated 18%–19% risk) will be bumped up or down to a different risk category because their estimate has now changed by 2%–3%, but these absolute changes, as discussed above, have little clinical relevance [13].

## Does Lowering hsCRP with Drugs Result in Improved Cardiovascular Outcomes?

In the JUPITER trial (Justification for the Use of Statins in Primary Prevention: An Intervention Trial Evaluating Rosuvastatin), rosuvastatin was shown to reduce the chance of developing a clinically important cardiovascular event. Table 2 outlines the impact of other drugs on hsCRP and cardiovascular events. Although each drug has various pharmacological effects the impact of lowering hsCRP with medication on cardiovascular events is consistently inconsistent.

**Table 2:** Examples of drugs that lower hsCRP and the impact these drugs have had on clinical outcomes.

Drug	Approximate % Decrease in hsCRP [References]	Effect on Clinical Outcomes [References]
Rosiglitazone	40 [23]	↑ [24]
Rofecoxib	↓^a^ [25],[26]	↑ [27]
Fibrates	30–85 [23]	↔ [28],[29]
Vitamin E	50–80 [23]	↑↔ [30],[31]
Niacin	25 [23]	↓ [32]
Ezetimibe	10 [33]	↔ [34]
Statins	15–50 [23]	↓ [35]

↑ Consistent evidence is available that the drug increases the risk of cardiovascular events.

↔ Evidence is incomplete or inconsistent as to the effect the drug has on cardiovascular events.

↓ Consistent evidence is available that the drug decreases the risk of cardiovascular events.

a Rofecoxib reduces hsCRP more than placebo but not enough data is provided in the referenced studies to give a specific % reduction.

## What Were the Outcomes of JUPITER?

The JUPITER investigators screened almost 90,000 people to enroll 17,802 who had an hsCRP≥2 mg/l and an LDL<3.4 mmol/l (130 mg/dl). Participants (mean age 66, median LDL of 2.8 mmol/l, average hsCRP of 4.2 mg/l) were randomized to rosuvastatin 20 mg daily or placebo. Based on the baseline characteristics of these participants, using the Reynolds risk score, the average participant in this trial would have had a 10-year risk of approximately 10%–15% [13]. The trial was stopped early, after a median follow-up of 1.9 years revealed that the combined endpoint of myocardial infarction, stroke, or death from cardiovascular causes occurred in 0.9% of participants taking rosuvastatin compared to 1.8% of patients taking placebo.

Ideally, the effects of hsCRP on risk assessment and treatment decisions would be evaluated using a randomized trial with two groups: one in which a risk assessment included hsCRP and one that did not. Then participants in each group would receive a drug known to improve cardiac outcomes at a predefined risk level. To date no trial, including JUPITER, has been designed to answer the question “Does the use of hsCRP in clinical practice result in reduced CVD outcomes and improved health?” A meta-analysis had already demonstrated that primary prevention with statins lowers the risk of CVD [18]. JUPITER might be considered unique in that the study participants had a reduced low-density lipoprotein (LDL) but ASCOT-LLA (Anglo-Scandinavian Cardiac Outcomes Trial–Lipid Lowering Arm) has already demonstrated that primary prevention patients at risk for CVD benefit from a statin despite a lower than “normal” LDL, albeit not as low an LDL as was evaluated in JUPITER (ASCOT-LLA mean LDL 3.4 mmol/l, JUPITER median LDL 2.8 mmol/l) [19]. It is important to remember that JUPITER was an evaluation of a fixed dose of rosuvastatin and thus getting subjects to “targets” is purely extrapolation, as none of the statin studies done to date, including the JUPITER trial, actually attempted to get subjects to a targeted cholesterol or hsCRP level [20]. The reductions in outcomes found in JUPITER were greater than those seen in other studies of statins in primary prevention. For example, a meta-analysis of primary prevention statin trials [18] found a 29.2% reduction in relative risk for major coronary events compared to 54% for the same outcome in JUPITER [1]. It is tempting to think the enrollment of patients with hsCRP≥2 mg/l contributed to the increased benefit seen in JUPITER. However, JUPITER was stopped early for benefit, and there is some suggestion that trials stopped early for benefit yield exaggerated treatment benefits [21]. Additionally, it is important to keep in mind the 54% reduction relative risk of major coronary events is actually a 0.2% per year absolute reduction (number need to treat of 500) [1].

## Is There Evidence That Baseline hsCRP Levels Predict Outcomes from Statin Therapy?

In a post-hoc analysis, Ridker et al. reviewed participants in the AFCAPS/TexCAPS study (Air Force/Texas Coronary Atherosclerosis Prevention Study). They stratified participants according to their baseline LDLs or hsCRPs (A, any hsCRP; H, LDL or hsCRP higher than the median; L, LDL or hsCRP lower than the median) to determine if different baseline levels of hsCRP or LDL could predict variations in the benefit of a statin. The main results are outlined in Table 3.

**Table 3:** AFCAPS/TexCAPS acute coronary disease results - Relative risks (and 95% CIs) associated with lovastatin therapy, according to baseline lipid and C-reactive protein levels.

LDL	CRP	Relative Risk of Acute Coronary Event	95% CI	5 Year Risk (%)
L	L	1.08	0.56–2.08	2.2
L	H	0.58	0.34–0.98	5.1
H	L	0.38	0.21–0.70	5.0
H	H	0.68	0.42–1.10	5.5
H	A	0.53	0.37–0.77	5.3
L	A	0.74	0.49–1.11	3.6

Data are from Table 2 in [36].

5 year risk (%), risk of an acute coronary event in the placebo arm group (in other words, this is a baseline risk for this population); A, any hsCRP; H, LDL or hsCRP higher than the median; L, LDL or hsCRP lower than the median.

Key issues to consider about the AFCAPS/TexCAPS data:

1. Only the L/L and the L/A groupings showed a difference in baseline 5-year risk.
2. In three of the categories (L/H, H/L, and H/A), lovastatin produced a reduction in events that was superior to placebo; however, owing to the overlapping CIs, no subset had a statistically different magnitude of outcome from any other subset.
3. Post-hoc analysis of subgroups defined after randomization are subject to a high risk of bias.

## Isn't There Evidence That Reducing Levels of hsCRP Predicts Outcomes?

The AFCAPS/TexCAPS retrospective evaluation was about what happened to subjects based on baseline LDL/hsCRP. Interestingly, no studies have actually looked prospectively at the question of getting patients to a target hsCRP or cholesterol. In the JUPITER study, a fixed-dose rosuvasatin trial, investigators did look at achieved (in contrast to AFCAPS/TexCAPS) hsCRP and LDL measurements to see if there was an association between achieved levels and outcome [22]. The categories they chose (above or below an LDL of 1.8, an hsCRP of 2 or 1) were prespecified. The key findings are outlined in Table 4.

**Table 4:** JUPITER results for cardiovascular events - Hazard ratios for incident cardiovascular events in JUPITER according to achieved concentrations of LDL cholesterol and hsCRP after initiation of rosuvastatin.

Dataset	LDL	CRP	Hazard Ratios for Incident Cardiovascular Events	95% CI	CVD Rate – Events per 100 Person Years
**Data for hsCRP cutoff<2 mg/l**	Placebo		1.0	NA	1.11
	H	H	1.06	0.72–1.55	1.11
	L	H	0.53	0.38–0.74	0.62
	H	L	0.42	0.18–0.94	0.54
	L	L	0.35	0.23–0.54	0.38
**Data for hsCRP cutoff<1 mg/l**	Placebo		1.0	NA	1.11
	H	H	0.89	0.62–1.28	0.95
	L	H	0.49	0.37–0.66	0.56
	H	L	0.46	0.11–1.85	0.64
	L	L	0.21	0.09–0.51	0.24

Data are from Table 4 in [22].

CVD rate, events rates per 100 person-years (the placebo numbers are the baseline risk in this population and the other numbers are the on treatment risk); H, LDL≥1.8 or hsCRP higher than 2 or 1; L, LDL<1.8 or hsCRP lower than 2 or 1.

Key issues to consider about the JUPITER data:

1. While some information about baseline characteristics was provided, none was provided for any of the subsets in Table 4 other than the low LDL/low hsCRP.
2. No information is provided regarding how much (relatively) LDL and hsCRP went down in each of the LDL/CRP subsets.
3. Participants who took 20 mg of rosuvastatin and did not achieve an LDL<1.8 or hsCRP<1 (H/H) experienced no clinical benefit. While we are not necessarily endorsing the approach, if one were to follow this evidence, patients who do not attain an LDL<1.8 and a hsCRP of less than 1 or 2 while on 20 mg of rosuvastatin should stop taking the drug as they will not derive a clinical benefit.
4. There is little difference in the results among participants who achieved hsCRP of <1 mg/dl or <2 mg/d, which suggests that as long as you get the hsCRP below 2 mg/dl any further reduction yields no additional benefit.
5. Participants who achieved both an LDL<1.8 and an hsCRP<2 (L/L), had a lower point estimate of benefit than participants who only achieved one of these breakpoints. The authors state that overall there was a *p*-value for trend across LDL cholesterol and/or hsCRP strata. However, the CIs for a number of the groups clearly overlap to a degree that does not allow one to draw specific conclusions about the differences in benefit between specific subsets.
6. It is unknown if lower LDL/hsCRP levels were attained due to differences in adherence to rosuvastatin, a factor that might help explain why the group that did not achieve specific LDL and hsCRP levels did not appear to benefit from statin therapy.

## Conclusion

The substantial intra-subject variation in hsCRP measurements makes it virtually impossible to assess the impact a therapy has on hsCRP in an individual patient. Even if the intra-subject variation is ignored, when hsCRP is used in addition to other established risk factors, the size of the absolute changes in risk estimates, even at the extremes of hsCRP levels, are unlikely to change an individual's decision to seek therapy. Finally, the evidence that cardiovascular events are reduced when a patient takes a drug that lowers hsCRP is inconsistent at best. Armed with this information, we hope clinicians can determine for themselves whether measuring hsCRP is an important part of a comprehensive risk profile or a clinically redundant practice.

## Abbreviations

CHD: coronary heart disease
CI: confidence interval
CVD: cardiovascular disease
HDL: high-density lipoprotein
hsCRP: high-sensitivity C-reactive protein
LDL: low-density lipoprotein
SBP: systolic blood pressure

## References

[1] Ridker PM, Danielson E, Fonseca FA, Genest J, Gotto AM (2008). Rosuvastatin to prevent vascular events in men and women with elevated C-reactive protein.. N Engl J Med.

[2] Woloshin S, Schwartz LM (2005). Distribution of C-reactive protein values in the United States.. N Engl J Med.

[3] Mitka M (2003). Panel endorses limited role for CRP tests.. JAMA.

[4] Grundy SM, Hansen B, Smith SC,, Cleeman JI, Kahn RA (2004). Clinical management of metabolic syndrome: report of the American Heart Association/National Heart, Lung, and Blood Institute/American Diabetes Association conference on scientific issues related to management.. Circulation.

[5] Bard RL, Rubenfire M, Eagle K, Clarke NS, Brook RD (2005). Utility of C-reactive protein measurement in risk stratification during primary cardiovascular disease prevention.. Am J Cardiol.

[6] Ridker PM, Paynter NP, Rifai N, Gaziano JM, Cook NR (2008). C-reactive protein and parental history improve global cardiovascular risk prediction: the Reynolds Risk Score for men.. Circulation.

[7] Ockene IS, Matthews CE, Rifai N, Ridker PM, Reed G (2001). Variability and classification accuracy of serial high-sensitivity C-reactive protein measurements in healthy adults.. Clin Chem.

[8] Macy EM, Hayes TE, Tracy RP (1997). Variability in the measurement of C-reactive protein in healthy subjects: implications for reference intervals and epidemiological applications.. Clin Chem.

[9] Campbell B, Badrick T, Flatman R, Kanowski D (2002). Limited clinical utility of high-sensitivity plasma C-reactive protein assays.. Ann Clin Biochem.

[10] Folsom AR, Chambless LE, Ballantyne CM, Coresh J, Heiss G (2006). An assessment of incremental coronary risk prediction using C-reactive protein and other novel risk markers: the atherosclerosis risk in communities study.. Arch Intern Med.

[11] Wang TJ, Gona P, Larson MG, Tofler GH, Levy D (2006). Multiple biomarkers for the prediction of first major cardiovascular events and death.. N Engl J Med.

[12] Shah T, Casas JP, Cooper JA, Tzoulaki I, Sofat R (2009). Critical appraisal of CRP measurement for the prediction of coronary heart disease events: new data and systematic review of 31 prospective cohorts.. Int J Epidemiol.

[13] (2009). Reynold's Risk Score.. http://www.reynoldsriskscore.org/.

[14] Anderson KM, Odell PM, Wilson PW, Kannel WB (1991). Cardiovascular disease risk profiles.. Am Heart J.

[15] Reynolds TM, Twomey P, Wierzbicki AS (2002). Accuracy of cardiovascular risk estimation for primary prevention in patients without diabetes.. J Cardiovasc Risk.

[16] Cook NR, Buring JE, Ridker PM (2006). The effect of including C-reactive protein in cardiovascular risk prediction models for women.. Ann Intern Med.

[17] Wilson PWF, Pencina M, Jacques P, Selhub J, D'Agostino R (2008). C-Reactive Protein and Reclassification of Cardiovascular Risk in the Framingham Heart Study.. Circ Cardiovasc Qual Outcomes.

[18] Baigent C, Keech A, Kearney PM, Blackwell L, Buck G (2005). Efficacy and safety of cholesterol-lowering treatment: prospective meta-analysis of data from 90,056 participants in 14 randomised trials of statins.. Lancet.

[19] Sever PS, Dahlof B, Poulter NR, Wedel H, Beevers G (2003). Prevention of coronary and stroke events with atorvastatin in hypertensive patients who have average or lower-than-average cholesterol concentrations, in the Anglo-Scandinavian Cardiac Outcomes Trial–Lipid Lowering Arm (ASCOT-LLA): a multicentre randomised controlled trial.. Lancet.

[20] (2009). Theheart.org.. http://www.theheart.org/editorial-program/926043.do.

[21] Montori VM, Devereaux PJ, Adhikari NK, Burns KE, Eggert CH (2005). Randomized trials stopped early for benefit: a systematic review.. JAMA.

[22] Ridker PM, Danielson E, Fonseca FA, Genest J, Gotto AM (2009). Reduction in C-reactive protein and LDL cholesterol and cardiovascular event rates after initiation of rosuvastatin: a prospective study of the JUPITER trial.. Lancet.

[23] Prasad K (2006). C-reactive protein (CRP)-lowering agents.. Cardiovasc Drug Rev.

[24] Singh S, Loke YK, Furberg CD (2007). Long-term risk of cardiovascular events with rosiglitazone: a meta-analysis.. JAMA.

[25] Monakier D, Mates M, Klutstein MW, Balkin JA, Rudensky B (2004). Rofecoxib, a COX-2 inhibitor, lowers C-reactive protein and interleukin-6 levels in patients with acute coronary syndromes.. Chest.

[26] Bogaty P, Brophy JM, Noel M, Boyer L, Simard S (2004). Impact of prolonged cyclooxygenase-2 inhibition on inflammatory markers and endothelial function in patients with ischemic heart disease and raised C-reactive protein: a randomized placebo-controlled study.. Circulation.

[27] McCormack JP, Rangno R (2002). Digging for data from the COX-2 trials.. CMAJ.

[28] Keech A, Simes RJ, Barter P, Best J, Scott R (2005). Effects of long-term fenofibrate therapy on cardiovascular events in 9795 people with type 2 diabetes mellitus (the FIELD study): randomised controlled trial.. Lancet.

[29] (2000). Secondary prevention by raising HDL cholesterol and reducing triglycerides in patients with coronary artery disease: the Bezafibrate Infarction Prevention (BIP) study.. Circulation.

[30] Vivekananthan DP, Penn MS, Sapp SK, Hsu A, Topol EJ (2003). Use of antioxidant vitamins for the prevention of cardiovascular disease: meta-analysis of randomised trials.. Lancet.

[31] Bjelakovic G, Nikolova D, Gluud LL, Simonetti RG, Gluud C (2007). Mortality in randomized trials of antioxidant supplements for primary and secondary prevention: systematic review and meta-analysis.. JAMA.

[32] (1975). Clofibrate and niacin in coronary heart disease.. JAMA.

[33] Sager PT, Capece R, Lipka L, Strony J, Yang B (2005). Effects of ezetimibe coadministered with simvastatin on C-reactive protein in a large cohort of hypercholesterolemic patients.. Atherosclerosis.

[34] Kastelein JJ, Akdim F, Stroes ES, Zwinderman AH, Bots ML (2008). Simvastatin with or without ezetimibe in familial hypercholesterolemia.. N Engl J Med.

[35] Kearney PM, Blackwell L, Collins R, Keech A, Simes J (2008). Efficacy of cholesterol-lowering therapy in 18,686 people with diabetes in 14 randomised trials of statins: a meta-analysis.. Lancet.

[36] Ridker PM, Rifai N, Clearfield M, Downs JR, Weis SE (2001). Measurement of C-reactive protein for the targeting of statin therapy in the primary prevention of acute coronary events.. N Engl J Med.

